# Vitamin K Supplementation for the Prevention of Cardiovascular Disease: Where Is the Evidence? A Systematic Review of Controlled Trials

**DOI:** 10.3390/nu12102909

**Published:** 2020-09-23

**Authors:** Caitlyn Vlasschaert, Chloe J. Goss, Nathan G. Pilkey, Sandra McKeown, Rachel M. Holden

**Affiliations:** 1 Department of Medicine, Queen’s University, Kingston, ON K7L 3V6, Canada; caitlyn.vlasschaert@queensu.ca (C.V.); chloeejuliet@gmail.com (C.J.G.); pilkeyn10@gmail.com (N.G.P.); 2 Bracken Health Sciences Library, Queen’s University, Kingston, ON K7L 2V5, Canada; sandra.mckeown@queensu.ca; 3 Department of Biomedical and Molecular Sciences, Queen’s University, Kingston, ON K7L 3V6, Canada

**Keywords:** vitamin K, cardiovascular disease, vascular calcification, arterial stiffness, atherosclerosis, matrix Gla protein

## Abstract

Matrix gla protein (MGP) is an important vitamin K-dependent inhibitor of vascular calcification. High levels of uncarboxylated, dephosphorylated MGP have been associated with vascular calcification and are responsive to vitamin K treatment. In this systematic review, we summarize the available evidence examining whether vitamin K supplementation improves surrogate measures of cardiovascular disease including artery and valve calcification, atherosclerosis and artery stiffening. Data from controlled trials of adults were obtained by searching Ovid MEDLINE, Embase, the Cochrane Central Register of Controlled Trials and the Web of Science Core Collection. We identified nine randomized controlled trials for review, including trials of vitamin K_1_ or vitamin K_2_ supplementation, that assessed a surrogate measure of cardiovascular disease including arterial calcification, atherosclerosis or arterial stiffening. For each trial, the risk of bias was assessed applying Cochrane Collaboration methodology. The findings indicate that vitamin K does not consistently prevent progression of calcification, atherosclerosis or arterial stiffness. There may be some benefit in people with calcification at study entry. Studies were heterogenous, with relatively short follow-up and outcome measures were varied. While vitamin K supplementation clearly improves the carboxylation of dephosphoylated MGP, its role in mitigating vascular calcification is uncertain, based on current evidence.

## 1. Introduction

Cardiovascular disease (CVD) is the leading cause of morbidity and mortality globally. Calcification of arteries and valves increases the risk for CVD and death in the aging population. Calcification occurs frequently in people with chronic kidney disease (CKD) and diabetes mellitus, where it is an independent risk factor for cardiovascular mortality [1]. Though traditional cardiac risk factors, such as hypertension and dyslipidemia, are common in these populations, they do not completely account for this increased risk.

Matrix Gla protein (MGP) is a vitamin K-dependent protein (VKDP) that counteracts vascular mineralization. MGP is highly expressed in calcifying arteries where it is thought to inhibit extra-osseous calcification by binding to growing hydroxyapatite crystals and inhibiting BMP-2 [2]. In order to be activated, vitamin K-dependent ϒ-carboxylation of the Gla residues is required. In addition to carboxylation, MGP is also phosphorylated post-translationally. In the setting of vitamin K deficiency or antagonism, the post-translational carboxylation of vitamin K-dependent proteins is impaired, resulting in increasing inactive fractions of these proteins in circulation [3]. Vitamin K deficiency is common in CKD [4] and ESKD [5], particularly in those on vitamin K antagonist medications such as warfarin [3]. In previous studies, each 100-point increment in dephosphorylated, uncarboxylated (dp-uc) MGP (in pmol/L) correlated roughly with a 10% increase in vascular calcification scores. Further, observational studies have suggested an association between dietary intake of vitamin K as well as biomarkers of vitamin K and reduced arterial calcification [6,7,8,9]. Thus, there is rationale to suggest that correcting vitamin K deficiency by supplementation with different forms of vitamin K may decrease calcification and improve CVD outcomes. We performed a systematic review of clinical trials that examined whether vitamin K supplementation influences surrogate measures of cardiovascular disease such as vascular calcification, vascular stiffness and intimal media thickness.

## 2. Materials and Methods

This systematic review was conducted according to the guidelines for Preferred Reporting Items for Systematic Reviews and Meta-Analyses [10]. The review question, formulated according to PICOS (Population, Intervention, Comparison, Outcomes and Settings) criteria (Table 1) was as follows: In randomized trials, does vitamin K supplementation influence surrogate markers of cardiovascular disease?

### 2.1. Eligibility Criteria

The following inclusion criteria were applied to obtain the selected studies: parallel randomized controlled trials of adult participants of any ethnicity that involved vitamin K supplementation with a control group (placebo or no-treatment control group) for a period of >12 weeks and measured a surrogate outcome related to cardiovascular disease at baseline and study end. Trials published up until April, 2020 were considered.

### 2.2. Intervention Types

Studies were restricted to >12 weeks in duration. All studies in which participants received a vitamin K supplement—either vitamin K_1_ (VK_1_, phylloquinone) or vitamin K_2_ (VK_2_, menaquinone-7, or MK-7)—compared with a non-supplemented group were considered for inclusion. Co-interventions were permissible if they were received by both groups.

### 2.3. Types of Outcome Measures

For the assessment of the impact of vitamin K treatment on CVD, data from any clinical trial that included a surrogate measure of CVD was considered. Surrogate measures of CVD included an image-based quantification of calcification burden or ultrasound assessment of atherosclerosis or arterial stiffness. Calcification was measured by computed tomography (CT) or positron emission tomography (PET) and was assessed in coronary arteries, aorta, femoral arteries and cardiac valves. Atherosclerosis was assessed by carotid intima media thickness (CIMT). Arterial stiffness was assessed by ultrasound-based measurement of pulse wave velocity (PWV) between the carotid and femoral arteries (cfPWV), as well as the carotid and radial arteries (crPWV). We excluded studies reporting only laboratory parameters. The primary outcome was the change in the measurement of the surrogate marker from baseline to study end. Secondary outcomes assessed within each of the identified trials included any additional surrogate measures of CVD that were assessed in addition to the primary outcome, as well as changes in MGP carboxylation and adverse effects.

### 2.4. Search Methods for Identification of Studies

A three-step search strategy was utilized to locate published studies for this review. An initial limited search of Ovid MEDLINE was executed, followed by an analysis of eligible studies to identify relevant text words contained in the title, abstract and author provided keywords, and the subject headings assigned to the article. A second search using all relevant text words and subject headings was then executed in Ovid MEDLINE and subsequently adapted for the other databases (Appendix A). The following databases were searched from inception in April 2020: Ovid MEDLINE, Embase, the Cochrane Central Register of Controlled Trials (CENTRAL), and the Web of Science Core Collection. No language or date restrictions were applied. The total number of records from all databases was 7668. After removing duplicate references in Covidence systematic review software, the total number of records screened was 5255. Bibliographies of included studies were reviewed for further references to relevant trials and abstracts from the last 5 years of the American Society of Nephrology Kidney Week and the annual meeting of the European Renal Association-European Dialysis and Transplant Association were hand-searched.

### 2.5. Study Selection

Two authors (NP, CG) independently screened all abstracts identified from the literature search. Those that were clearly irrelevant were excluded. Full texts of remaining studies were obtained, screened and excluded if irrelevant. Disagreements were resolved by discussion or with a third reviewer (RH).

### 2.6. Data Items

The following data was extracted by two independent reviewers (CV, RH): study design, inclusion and exclusion criteria for patients, method for assigning patients to different treatment groups, details of the intervention and control arms, number of patients randomized, lost to follow-up and analyzed, demographic and clinical information for patients including age and sex and important co-morbidities, and types of surrogate measures of cardiovascular disease. No formal assessment of agreement between raters was obtained. In addition, we obtained the first author’s name, year of publication, funding source and source of vitamin K. We extracted the net change in the surrogate measure of CVD before and after vitamin K treatment as the main outcome measure.

### 2.7. Data Synthesis and Analysis

Data was synthesized in a narrative form and then sub-grouped based on the different surrogate outcome measures of CVD (e.g., arterial calcification, atherosclerosis (CIMT) and arterial stiffness (PWV)). Summary tables and text provide study descriptions and clinical characteristics. The over-arching goal was to summarize the direction of any observed effects of vitamin K across studies. Meta-analysis was not performed.

### 2.8. Assessment of Risk of Bias

Two review authors independently assessed the risk of bias for each individual study using the Cochrane Risk of Bias tool (NP, CG) [11]. Disagreements were resolved by discussion or with a third or fourth review author (CV, RH). The following six criteria were examined and the risk was assessed as low, high or unclear: adequate sequence generation, allocation concealment, blinding, incomplete data outcome, and selective reporting.

### 2.9. Measurement of Treatment Effect

All trials reported continuous measures at baseline and study end with tests of statistical significance to determine treatment effect. For the purposes of summarizing results, we categorized the trials according to the primary or secondary outcome measures reported. For example, some trials reported the impact of vitamin K treatment on more than one surrogate measure of CVD (e.g., coronary artery calcium (CAC) score and PWV).

## 3. Results

### 3.1. Study Selection

**Figure 1:**
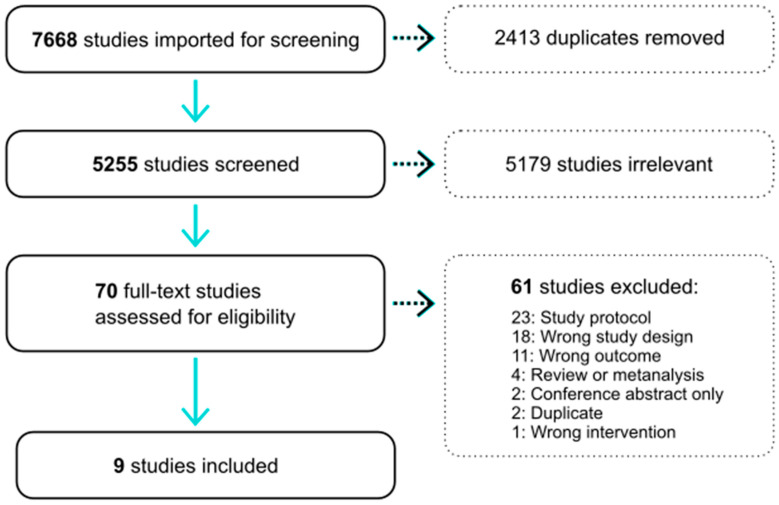
Flow chart depicting the literature search process.

The results of the bibliographic search are presented in Figure 1. Of the 7668 results from the first search, 2413 duplicates were removed. 5179 articles were excluded after abstract review. Of the 70 articles considered for data extraction, nine articles were ultimately included in this systematic review. Review of abstracts for the last 5 years of Kidney Week and the ERA-EDTA identified one additional clinical trial that is published in abstract form. However, we were not able to obtain further sufficient information to include this trial formally in our review. Of the included nine studies, all were controlled trials of a vitamin K treatment and included measurement/s of a surrogate measure of cardiovascular disease. In total, 1589 patients (745 treatment and 844 control) were included. Five trials studied healthy populations, three studied participants with low kidney function or on hemodialysis and one study was of people with type 2 diabetes. The participants were generally older with the mean age ranging from 55 to almost 80 years of age.

### 3.2. Study Characteristics

All nine studies selected for review were randomized trials published in English. Table 2 includes all studies (*n* = 9) in alphabetical order by the first author’s surname. The included studies involved 1589 participants. Seven trials were randomized and controlled whereas two were open-label. Only one trial was conducted outside of Europe and the majority of trials were conducted in a single-center. The intervention received in three trials was VK1 and in the remaining six trials was VK2. The dose of VK1 ranged from 500 mcg to 2 mg daily and the dose of VK2 ranged from 90 μg to 2000 μg daily. Follow-up ranged from 6 months to 3 years. The primary outcome was a measure of calcification in six studies and a measure of atherosclerosis in three studies. The timing of outcome measures was at baseline and study end.

### 3.3. Risk of Bias Assessment

Quality measures of the randomized trials are presented in Table 3. There was a low risk of bias across all parameters in the studies by Shea et al. and Zwakenberg et al. [19,20]. Otherwise, sequence generation and allocation concealment was deemed unclear if not reported and was high in one trial due to deviation from the intended intervention [18]. Two trials were at high risk of bias with regard to blinding of participants and personnel based on their open-label study design [13,14]. In the majority of cases, blinding of outcome assessors was deemed either low risk or unclear if it was not specifically reported. Four trials had either incomplete or unclear outcome data, whereas the risk of bias from selective outcome reporting was low in the majority of trials and unclear in two.

### 3.4. Vitamin K Supplementation and Measures of Artery Calcification

Six of the nine included trials examined the effect of vitamin K supplementation on the calcification of major vessels and cardiac valves (Table 4). Three studies examined changes in coronary artery calcification (CAC) scores. All three found no significant difference with vitamin K supplementation in their intention-to-treat analyses [14,17,19]. However, Shea et al. found a positive impact of vitamin K1 supplementation on attenuating CAC progression in two subgroups: those who were >85% adherent to the treatment protocol, and those who had pre-existing CAC (baseline Agatston scores ≥10) [19]. A smaller Polish trial (total *n* = 40) that enrolled only patients with pre-existing CAC found a trend toward improved CAC scores with MK-7 supplementation [17]. This finding was nearly significant (*p* = 0.06) when those with very high baseline CAC scores (>1000 AU) were excluded from the analysis (total *n* = 4 removed). A longitudinal study examining changes in CAC over 18 months in patients on HD with non-valvular atrial fibrillation (AF) using different anticoagulation strategies (rivaroxaban plus MK-7, rivaroxaban alone, warfarin alone) found no significant difference between its three arms [14]. Five studies examined calcification changes of components of the left heart outflow tract: mitral valve (*n* = 1), aortic valve (*n* = 2), thoracic aorta (*n* = 1), abdominal aorta (*n* = 1) and femoral arteries (*n* = 1). An open label trial that enrolled patients without CKD and with minimal aortic valve calcification (AVC) at baseline found that AVC progressed by 10% in patients taking 2 mg vitamin K1, compared to 22% for those taking placebo (*p* = 0.04) [13]. However, all other trials showed no benefit in terms of reduction in calcification with vitamin K supplementation. Calcification scores for both aortic and mitral valves as well as thoracic aorta did not significantly differ in the three-armed De Vriese et al. trial [14]. The Oikonomaki trial looked at the progression of abdominal aorta calcification scores in participants with ESKD and found no significant change after 12 months of MK-7 supplementation [18]. One trial compared changes in calcification of the femoral artery using standard imaging (CT scan) compared to ^18^F-NaF PET scan, which reportedly measures active calcification [20]. Interestingly, they found that while calcification scores by CT scan did not change with 6-months of MK-7 supplementation, ^18^F-NaF PET-CT scan showed increased calcification activity in the MK-7 group compared to placebo (0.25; 95% CI: −0.02, 0.51; *p* = 0.06). Overall, the role of vitamin K supplementation in modifying vascular calcification is unclear, especially considering the contrasting results obtained when using different imaging modalities.

### 3.5. Vitamin K Supplementation and Atherosclerosis

Change over time in CIMT was evaluated in three of the nine trials but was not the primary end-point for any of the studies [12,15,17] (Table 5). In total, 225 patients were included in these analyses. In a study of older people with established cardiovascular disease, 100 mcg of MK-7 daily for 6 months had no impact on CIMT compared to placebo [15]. One larger trial examined healthy post-menopausal women over 3-years [12]. One mg vitamin K_1_ per day study showed no benefit in attenuating CIMT progression over time. In a small study of participants with CKD, MK-7 and vitamin D resulted in an attenuated increase in CIMT over 9 months but this difference was not significant. Overall, the results of these trials do not support a role for vitamin K in modifying atherosclerosis when measured by CIMT.

### 3.6. Vitamin K Supplementation and Arterial Stiffness

Of the nine vitamin K treatment trials, three examined changes in PWV, and in one trial this was the primary end-point (Table 6). Fulton et al. randomized 80 elderly participants to either 100 μg of MK-7 daily versus placebo for 6 months [15]. In this placebo-controlled trial with minimal loss to follow-up, there was no impact of MK-7 treatment on change in PWV. However, there was a modest, non-significant improvement in PWV in the VK1-treated group once adjusted for baseline values. De Vriese et al. examined 2000 μg MK-7 supplementation in rivaroxaban-treated hemodialysis patients over 18 months [14]. Mixed modeling revealed that the change in PWV over time was not significantly different across treatment arms. In a 36 month trial conducted in healthy post-menopausal women, the absolute change in PWV over time was significantly attenuated in the MK-7 group at study end [16]. However, a different measure of arterial stiffness, stiffness index β, which comprised the arterial diameter and distension during diastole and blood pressure, demonstrated no significant between-group differences over the 3 years of treatment. Overall, the results of these trials do not consistently support a role for vitamin K in attenuating changes in arterial stiffness in both the general population and in higher risk groups, including the elderly and hemodialysis populations. However, there is significant heterogeneity between these three trials in terms of duration, study population and dose of MK-7. The one trial reporting a significant treatment effect was conducted over 3 years in a relatively low risk population, suggesting that duration may be an important consideration.

### 3.7. Vitamin K and Changes in MGP

Of the nine clinical trials included in this review, the response of dp-ucMGP to either vitamin K1 or MK-7 treatment was examined in eight of the studies and the change in total MGP was reported in two studies (Table 7). A significant treatment effect on the decrease in dp-ucMGP was observed in seven of eight trials. There was no impact of vitamin K treatment on total MGP levels.

### 3.8. Adverse Effects

Of the nine clinical trials included in this review, five reported the frequency and types of adverse events experienced by trial participants (Table 8). In three trials, no adverse events were experienced in either the treatment or control arm. There was a high prevalence of adverse events reported in the trial of elderly individuals [15]. There were more reported falls and gastrointestinal side effects observed in the vitamin K treatment group but no differences with regard to serious adverse events or death. In the De Vriese trial, no adverse events associated with vitamin K supplementation were reported. However, there were 36 major or life-threatening bleeding events reported in this trial of high risk participants receiving some form of anticoagulation. There was no report of thrombosis in any of the clinical trials.

## 4. Discussion

We performed a systematic review of trials that have been conducted to examine whether vitamin K supplementation mitigates cardiovascular disease progression by examining correlates of CVD including measured vascular calcification, intima-media thickness, and stiffness. While vitamin K supplementation improves vitamin K status, based on the carboxylation status of dpMGP, its role in mitigating vascular calcification is unclear, based on current evidence. Vitamin K supplementation does not appear to significantly affect atherosclerosis and the results derived from studies examining arterial stiffness are not consistent.

The mechanisms by which vascular calcification occur are multifactorial and becoming progressively clearer [21]. Local inflammation appears to prompt the formation of initial vascular microcalcifications and drives the ensuing progressive mineralization of multiple vessel wall layers [22]. Aberrances in vitamin D signaling and dysregulated phosphate metabolism are recognized to further promote vascular calcification in patients with CKD [23]. The vascular smooth muscle layer (the tunica media) is the most prominent calcification site in CKD but also occurs in diabetes and aging [24]). This pathologic process leads to increased vessel wall thickness and reduced elasticity. Matrix Gla protein, a vitamin K-dependent protein, inhibits this process. Intimal layer calcification, on the other hand, is associated with luminal atherosclerotic plaque formation.

Total vascular calcification is typically quantified using the Agatston score, which is a weighted calculation of macroscopic calcification densities observed on CT imaging [25]. While the Agatston score was developed to study coronary artery calcification (CAC) and is correlated with MACE in this context, its use has been extended to study other vessels [26]. Newer modalities can assess different properties of vascular calcification [27]. ^18^F-NaF PET scanning of arteries can detect microcalcifications and may be more sensitive for newly formed calcium deposits [28], whereas invasive imaging techniques such as angiography, with or without optical coherence tomography (OCT), offer greater spatial resolution of mineralization sites within and along the vessel walls [27].

Of the six trials that measured changes in calcification of major vessels or valves, five concluded that there was no significant benefit of vitamin K. Sub-analyses of two of these studies suggest there might be lowered CAC progression in patients with significant calcification at baseline (Agatston score ≥10) [17,19]. One study reported a benefit in its per-protocol analysis [13]. It examined 72 patients with minimal baseline calcification and without CKD but with relative vitamin K deficiency. Vitamin K1 supplementation was associated with a lower delta calcification volume of the aortic valve over 12 months (78 ± 165 µL vs. 181 ± 234 µL, *p* = 0.04), including when the data were indexed to BSA [13]. However, the risk of bias for this “proof-of-concept” study was high owing to its open-label design. In addition, there was a substantial drop-out rate (27%).

When using the CT scan-based Agatston score, CAC progression should be assessed over a minimum 1-year follow-up period in order to detect macroscropic changes in calcification [29,30]. A 2019 trial compared CT scan scores to scores obtained via a newer modality, ^18^F-NaF PET scanning, after 6 months of MK-7 supplementation [20]. They found no significant difference in femoral artery calcification mass at this time point on the CT scan. However, on the ^18^F-NaF PET scan, they saw that the target-to-background ratio (TBR) tended to increase in the MK-7 group compared with placebo, suggesting that there may be more ongoing active vascular calcification in the MK-7 treatment group. Resolution limits may therefore bias the assessment of arterial calcification scores via conventional imaging modalities such as CT scan. Taken together, in this review, we did not find conclusive evidence that vitamin K supplementation impedes vascular calcification.

Intimal calcification occurs in conjunction with atherosclerotic plaque formation and is associated with dyslipidemia, inflammation and other traditional Framingham risk factors. Ultrasound measurement of the carotid intima media thickness (CIMT) is one established surrogate marker of atherosclerosis burden [30]. CIMT is defined as the distance between the lumen–intima and the media–adventitial interfaces of a segment of the carotid artery and can reflect progression of atherosclerosis or non-atherosclerotic enlargement of the arterial wall. Variability in the measurement of CIMT makes it difficult to compare between studies or combine results from different studies [31]. Despite these limitations, many studies have demonstrated that CIMT is associated with the presence and severity of atherosclerosis and predicts cardiovascular events, as reviewed by Katakami et al. [32].

Dephosphorylated uncarboxylated MGP has been associated with CIMT in healthy post-menopausal women [16] but has not been similarly linked in higher risk populations including diabetes and CKD [33,34]. However, MGP has been detected in human atherosclerotic plaque [35] and antagonizing vitamin K with warfarin has been linked to increased atherosclerotic plaque [36]. Thus, there is rationale that vitamin K deficiency and/or vitamin K treatment could modify atherosclerosis. This concept has been tested in one pre-clinical study that used an atherosclerosis-prone mouse model. Vitamin K2 treatment (40 mg/kg/day) daily for 12 weeks inhibited intimal calcification of the aorta induced by a high-fat diet [37].

Our assessment of the three trials does not support a role for vitamin K in modifying atherosclerosis in humans when measured by CIMT. In the study of older people with established CVD, 100 mcg of MK-7 daily for 6 months had no impact on CIMT compared to placebo [15]. However, CIMT did not change appreciably in either the MK-7 group or the placebo group. It is possible that six months may be an insufficient duration of time to detect change. One larger trial examined healthy post-menopausal women and examined a longer treatment duration of 3 years and similarly showed no benefit of 1 mg of vitamin K_1_ per day in attenuating CIMT progression over time [12]. The approximated annual change in CIMT of between 0.007 and 0.017 mm/year reported by this study is similar to previous reports of expected annual CIMT change in healthy people (0.007–0.008 mm/year) and people with diabetes, where it increases to 0.034 mm/year (95% CI 0.029–0.039) [38]. In contrast, in a small group of patients with non-dialysis dependent CKD, CIMT increased significantly over approximately 9 months in both treatment and control arms [17]. However, the increase of 0.06 ±0.8 mm in the vitamin MK-7 + D group and 0.136 ± 0.05 mm in the vitamin D group exceeds that of previous reports in a high risk group. The authors suggested that there was a significant impact of MK-7 treatment on attenuating the increase in CIMT; however the analysis appeared only to have included a sub-set of the participants randomized to 90 μg of MK-7 daily. Taken together, despite the cross-sectional association between biomarkers of vitamin K status (dp-ucMGP) and CIMT that have been observed in healthy people, there is limited clinical trial evidence to support a treatment role for vitamin K to attenuate atherosclerosis in people at low-risk. The trials conducted in the higher risk populations were conducted over a relatively short period of time. It is conceivable that a longer duration of study or different dosages of vitamin K may be required in people at higher risk, including the elderly or people with CKD or diabetes, where medial calcification may be more likely to occur.

Medial calcification is characterized by hydroxyapatite deposition in the medial layer of the artery. This type of calcification has classically been associated with vascular smooth muscle cell conversion to osteoblast-like phenotype. It can occur in the absence of atherosclerosis and may involve the arterial tree diffusely. Clinical sequelae of vascular stiffening include hypertension and left ventricular hypertrophy. Measurement of pulse wave velocity (PWV) is a surrogate measure of vascular stiffness. The most validated method to assess vascular stiffness is based on the measurement of the velocity of pulse waves as they travel along the arterial tree between two sites most typically between the carotid and femoral artery. PWV increases with age and with hypertension and is linked to inflammation [39,40]. Higher PWV is predictive of poor outcomes including CV and all-cause mortality as well as stroke [41]. Arterial stiffness is worse in diabetic versus non-diabetic patients and worsens as kidney function declines, where it is linked to disorders of bone and mineral metabolism [39]. In patients with CKD, higher PWV predicts death, progression to ESKD as well as new-onset heart failure [42,43]. The use of PWV for monitoring the efficacy of cardiovascular treatments is plausible but not demonstrated.

The level of ucMGP has been consistently associated with higher PWV in studies of the general population, as reviewed by Roumeliotis et al. [44], whereas in studies that have examined patients with CKD, the findings have been less consistent. In one case, a control study of hemodialysis patients, those taking warfarin had a significantly greater increase in carotid-femoral PWV after 1 year compared to HD patients not taking warfarin [45]. In an open-label study of 26 participants, an 18% reduction in PWV was observed with MK-4 supplementation but only in the four people with vitamin K deficiency at baseline [46]. In an open-label, single-arm study of kidney transplant recipients, 360 mμg of MK-7 daily was associated with a 14.2% reduction in PWV at 8 weeks and the improvement was independently associated with the reduction in dp-ucMGP [47]. In a pre-clinical study of rats with CKD, warfarin treatment significantly increased aortic PWV, but there was no difference in PWV observed in rats treated with 4 weeks of either a low vitamin K1 diet or a high vitamin K1 diet [48].

Of the nine vitamin K treatment trials, three examined changes in PWV, with one as the primary end-point for the trial. MK-7 was not beneficial in either elderly people or participants with non-dialysis dependent CKD over 6 to 12 months [15]. De Vriese et al. examined 2000 μg MK-7 supplementation in rivaroxiban-treated hemodialysis patients over 18 months [14]. This trial attempted to answer an important question with direct clinical implications for the management of patients with ESKD who require anticoagulation. Mixed modeling revealed that the change in PWV over time was not significantly different across treatment arms. The main limitation of this study was the small sample size, the loss to follow-up over time and the substantial burden of CVD at baseline, highlighting some of the difficulties in conducting longitudinal trials in such a high-risk population. Further, the reduction in dp-ucMGP was similar in rivaroxaban-treated patients regardless of MK-7 supplementation, suggesting that the 2000 μg dose of MK-7 may be insufficient, at least in patients with ESKD. In a 36 month trial conducted in healthy post-menopausal women, the change in the absolute value of PWV over time was different between the MK-7 and placebo groups at study end [16]. A different measure of arterial stiffness, stiffness index β, which comprised the arterial diameter and distension during diastole and blood pressure, also demonstrated no significant between group differences over the 3 years of treatment. By stratifying participants into higher and lower aortic stiffness at baseline, stiffness index β decreased with MK-7 treatment; however, the distribution of the sample size and follow-up for these analyses is uncertain. The K4Kidneys trial, published to date in abstract form only, included 160 Scottish participants with CKD stage 3 and 4. Those randomized to 400 μg of vitamin K2 daily showed no difference in PWV at 12 months compared to placebo. Overall, the human clinical trial evidence does not uniformly support a beneficial effect of vitamin K on measures of arterial stiffness in elderly or higher risk populations. A significant benefit was noted in the one trial with longer treatment duration.

The level of dp-ucMGP has been associated with several adverse cardiovascular risk factors, including obesity and hypertension, and predicts cardiovascular events in the general population [49,50]. Cross-sectional studies indicate that the level of dp-ucMGP also depends on the total amount of MGP present; this is evident in the studies of CKD patients where all fractions of MGP are typically higher [6]. Thus, it is conceivable that MGP and its fractions in circulation may be related to the presence of CVD. Although it is evident from the trials included in this review that dp-ucMGP responds to vitamin K supplementation, whether improving dp-ucMGP translates to differences in outcomes, including vascular calcification, appears less certain. The highest quality RCT to date, involving 574 healthy participants, supports this line of thinking. In a post-hoc analysis of the trial by Shea et al., vitamin K1 supplementation significantly reduced dp-ucMGP levels; however, the change in dp-ucMGP did not correlate with the change in CAC score [51] Although the majority of studies included in our review examined changes in dp-ucMGP in response to vitamin K treatment, none reported whether the change dp-ucMGP correlated with changes in the outcome measure. For example, in the open-label study of participants with mild AS, vitamin K1 reduced both dp-ucMGP and aortic valve calcification progression [13]. However, whether the attenuation in valve calcification correlated with changes in dp-ucMGP was not reported. Similarly, MK-7 supplementation reduced arterial stiffness in a sub-set of women with high stiffness at baseline and resulted in substantial reductions in dp-ucMGP, but whether this reduction correlated with attenuation of stiffness progression was similarly not reported [16].

One pharmaceutical company, Nattopharma, was the source of vitamin K for five of the nine clinical trials. Three trials were conducted with the support of federal or national-based health society funds, three trials were conducted with industry funding, two trials were conducted using university-level funding and one trial received no funding. We did not observe any clear patterns related to source of funding or vitamin K with regards to study outcomes and conclusions.

The studies included in this review used varying doses of vitamin K. We therefore cannot rule out that a beneficial effect might be observed if higher doses were given, particularly in high risk groups. Two trials currently being conducted in hemodialysis patients use substantially higher doses of vitamin K1 than have been previously studied [52,53]. Although the results do not uniformly indicate a vitamin K treatment benefit, analysis of sub-groups with baseline calcification or per-protocol analyses suggest that there may be benefit in those with pre-existing disease who adhere to treatment. Taken together, there is presently a lack of well-conducted and adequately powered randomized controlled trials in this research field.

## 5. Conclusions

There is presently a lack of randomized trial evidence to support a beneficial role for vitamin K in preventing the worsening of surrogate measures of CVD. The trials conducted to date are primarily small, single-center trials and are heterogeneous with regard to type of vitamin K administered, dose of vitamin K, population studied and outcome measures. However, an alternative explanation may be that abnormalities in dp-ucMGP, which are sensitive to vitamin K supplementation, may be a consequence of CVD. Our assessment is that an improvement in surrogate measures of CVD with vitamin K supplementation has not been consistently demonstrated in the clinical trials to date and no clinical trial has examined important clinical events including mortality. In those trials that demonstrate a vitamin K treatment effect on a surrogate measure of CVD, the causal pathway linking the benefit to an improvement in dp-ucMGP has not been demonstrated. At present, it is not clear from human clinical trials that a causal pathway exists between vitamin K and reduced cardiovascular end-points that operates through enhanced MGP carboxylation and is responsive to vitamin K supplementation.

## Figures and Tables

**Table 1:** PICOS (Population, Intervention, Comparison, Outcomes and Settings) criteria for the inclusion of studies evaluating the effects of vitamin K supplementation on vascular properties.

Parameter	Inclusion Criteria
Population	Adult
Intervention	Controlled Vitamin K (K1 or K2) intake
Comparison	Non-exposed control group
Outcome	Any measurement of vascular calcification, vascular stiffness, vascular flow limitations (e.g., intimal media thickness, pulse wave velocity)
Setting	Randomized controlled trials

**Table 2:** Study characteristics of included trials.

Author, Year, Country	Study Design	Number of Centers	Population	Intervention	Control	Primary Outcome	Randomized (*n*)		Source of Vitamin K	Length of Follow Up (Months)	Age (SD)	
							Vitamin K	Control			Vitamin K	Control
Braam, 2003, Netherlands [12]	RCT	1	Community	Vitamin K_1_ 1 mg, daily; Multivitamin ^1^	Multivitamin ^1^	Carotid Vessel Wall Characteristics	66	61	INFECTOPHARM, USA	36	55.4 (2.8)	55.9 (2.8)
					Placebo			61				54.1 (3.0)
Brandenburg, 2017, Germany [13]	Open Label	1	Community	Vitamin K_1_, 2 mg daily	Placebo	AVC Volume Score	50	49	Nattopharma, Norway	12	NR^3^	NR
De Vriese, 2020, Belgium [14]	RCT	3	Hemodialysis	MK-7, 2000 μg thrice weekly + 10 mg Rivaroxaban daily	10 mg rivaroxaban daily	Change in Calcification Scores	42	46	Nattopharma, Norway	36	79.6 * (73.2–83.1)	79.9 (74.4–83.9)
					VKA			44				80.3 (71.5–84.3)
Fulton, 2015, Scotland [15]	RCT	2	Community	MK-7, 100 μg daily	Placebo	FMD	40	40	Nattopharma, Norway	6	76.0 (4.4)	77.1 (4.8)
Knapen, 2015, Netherlands [16]	RCT	1	Community	MK-7, 180 μg daily	Placebo	Carotid artery Stiffness Index β	120	124	Nattopharma, Norway	36	59.8 (3.5)	59.3 (3.1)
Kurnatowska, 2015, Poland [17]	RCT	1	CKD	MK-7 90 μg; Vitamin D 10 μg; daily	Vitamin D, 10 μg; daily	CAC Score Change	29	13	Nattopharma, Norway	9	NR	NR
Oikonomaki, 2019, Greece [18]	Open Label	1	Hemodialysis	MK-7, 200 μg daily	Standard Care	CAC Score	44	58	Solgar, USA	12	70.18 (12.48)	66.65 (16.4)
Shea, 2009, United States of America [19]	RCT	1	Community	Vitamin K_1_, 500 μg daily; Multivitamin ^2^	Multivitamin ^2^	Change in CAC Score	238	236	Hermes Arzeneimittel GmbH, Germany	36	68 (6)	68 (5)
Zwakenberg, 2019, Netherlands [20]	RCT	1	Type 2 Diabetes	MK-7, 360 μg daily	Placebo	Target to Background Ratio of ^18^F-NaF PET Uptake	35	33	Nattopharma, Norway	6	69.1 (8.4)	69.1 (8.4)

* Age presented as mean ± SD or median (IQR); ^1^ Multivitamin containing calcium 500 mg, zinc 10 mg, magnesium 150 mg, vitamin D_3_ 8 μg; ^2^ Multivitamin containing calcium 600 mg, Vitamin D 400 IU; Abbreviations: RCT—randomized controlled trial; AVC—aortic valve calcification; NR—Not Reported; MK—menaquinone;VKA—vitamin K antagonist; FMD—flow-mediated dilation; CKD—chronic kidney disease; CAC—coronary artery calcification; PET—positron emission tomography.

**Table 3:** Study quality assessment.

	Sequence Generation	Allocation Concealment	Blinding of Participants and Personnel for All Outcomes	Blinding of Outcome Assessors for All Outcomes	Incomplete Outcome Data for All Outcomes	Selective Outcome Reporting
Braam [12]	low	low	low	low	high	low
Brandenburg [13]	unclear	unclear	high	low	low	low
DeVriese [14]	low	low	high	low	low	low
Fulton [15]	low	low	low	low	unclear	low
Kurnatowska [17]	low	unclear	low	low	low	low
Shea [19]	low	low	low	low	low	low
Zwakenberg [20]	low	low	low	low	low	low
Knapen [16]	unclear	unclear	low	unclear	unclear	unclear
Oikonomaki [18]	high	unclear	high	unclear	high	unclear

**Table 4:** Effect of vitamin K supplementation on calcification outcomes.

Author, Analysis Conducted	Follow-up Period (mos)	Study Arms	Age (Mean ± SD or Median (IQR))	Number of Patients Randomized (*n*)	Number Lost to Follow-up (*n*)	Number Included in Final Analysis (*n*)	Outcome Metric	Location of Measurement	Outcome Measurements		*p* Value for Effect
									Baseline	End of Follow Up	
De Vriese [14]	18	Warfarin (target INR 2–3)	80.3 (71.5–84.3)	44	27	44	Median (IQR) percentage change in Agatston score (AU)	Coronary Artery	1816 (461–3000)	+29.4% (4.0–47.6%)	0.43
		Rivaroxaban 10 mg daily	79.9 (74.4–83.9)	46	22	46			1611 (427–3078)	+19.8% (7.2–37.9%)	
		Rivaroxaban 10 mg daily plus MK-7 2 mg thrice weekly	79.6 (73.2–83.1)	42	20	42			1465 (155–2755)	+18.6% (7.2–110.0%)	
		Warfarin (target INR 2–3)	80.3 (71.5–84.3)	44	27	44		Thoracic Aorta	11,566 (5722–18,193)	+19.9% (4.7–56.9%)	0.79
		Rivaroxaban 10 mg daily	79.9 (74.4–83.9)	46	22	46			8991 (4165–22,185)	+18.7% (4.6–48.8%)	
		Rivaroxaban 10 mg daily plus MK-7 2 mg thrice weekly	79.6 (73.2–83.1)	42	20	42			7864 (4135–14,109)	+25.6% (8.2–61.4%)	
		Warfarin (target INR 2–3)	80.3 (71.5–84.3)	44	27	44		Aortic and mitral valves (total)	689 (222–1391)	+57.1% (23.0–98.6%)	0.81
		Rivaroxaban 10 mg daily	79.9 (74.4–83.9)	46	22	46			415 (71–2276)	+33.4% (5.8–84.2%)	
		Rivaroxaban 10 mg daily plus MK-7 2 mg thrice weekly	79.6 (73.2–83.1)	41	20	42			339 (40–1028)	+36.3% (3.1–132.6%)	
De Vriese [19]	18	Warfarin (target INR 2–3)	80.3 (71.5–84.3)	44	27	44	Median (IQR) percentage change in calcification volume (mm^3^)	Coronary Artery	632 (249–1215)	+25.1% (10.6–44.7%)	0.43
		Rivaroxaban 10 mg daily	79.9 (74.4–83.9)	46	22	46			647 (195–1199)	+14.9% (2.7–34.0%)	
		Rivaroxaban 10 mg daily plus MK-7 2 mg thrice weekly	79.6 (73.2–83.1)	42	20	42			548 (108–993)	+29.3% (9.7–56.0%)	
		Warfarin (target INR 2–3)	80.3 (71.5–84.3)	44	27	44		Thoracic Aorta	3636 (1672–5432)	+24.9% (8.8–46.6%)	0.62
		Rivaroxaban 10 mg daily	79.9 (74.4–83.9)	46	22	46			2930 (1352–6244)	+15.6% (5.1–35.0%)	
		Rivaroxaban 10 mg daily plus MK-7 2 mg thrice weekly	79.6 (73.2–83.1)	42	20	42			2834 (1478–4541)	+19.5% (7.3–56.0%)	
		Warfarin (target INR 2–3)	80.3 (71.5–84.3)	44	27	44		Aortic and mitral valves (total)	239 (111–446)	+43.9% (15.6–77.1%)	0.33
		Rivaroxaban 10 mg daily	79.9 (74.4–83.9)	46	22	46			175 (43–660)	+15.4% (−5.6–44.3%)	
		Rivaroxaban 10 mg daily plus MK-7 2 mg thrice weekly	79.6 (73.2–83.1)	42	20	42			139 (37–430)	+37.7% (12.1–109.9%)	
Brandenburg [13]	12	Vitamin K1 2 mg daily	69.3 ± 10.1	50	12	38	Mean calcification volume ± SD (mL)	Aortic Valve	793 ± 742	871 ± 791	0.04
		Placebo control group	68.9 ± 7.9	49	15	34			836 ± 856	1017 ± 939	
Kurnatowska [17]	9	MK-7 90 µg daily plus Vitamin D	not reported	29	1	28	Mean change ± SD in CAC Score (AU)	Coronary Artery	58.1 ± 106.5		0.7
		Vitamin D Control		13	1	12			74.4 ± 127.1		
		MK-7 90 µg daily plus Vitamin D		29	1	28	Median change (IQR) in CAC Score (AU)		11.0 (0–55.5)		0.2
		Vitamin D Control		13	1	12			20.5 (8.0–119.0)		
Oikonomaki [18]	12	MK-7 200 µg daily	70.09 ±12.68	44	22	22	Mean ± SD aortic calcification volume (mm^3^)	Abdominal Aorta	6343.29 ± 4176.29	8128.64 ± 5534.46	NS
		Control	64.74 ±16.95	58	28	30			6529.25 ± 4689.64	8609.25 ± 6781.74	
		MK-7 200 µg daily	70.09 ±12.68	44	22	22	Mean ± SD aortic calcification mass (gr)		2394.42 ± 1905.12	3009.51 ± 2446.57	NS
		Control	64.74 ±16.95	58	28	30			2914.27 ± 3786.69	3557.06 ± 3033.08	
		MK-7 200 µg daily	70.09 ±12.68	44	22	22	Mean ± SD aortic calcification score (Hounsfield Units		7827.88 ± 5493.38	10,412.53 ± 7227.2	NS
		Control	64.74 ±16.95	58	28	30			8253 ± 6298.94	11,036.58 ± 9053.24	
Shea [19]	36	Vitamin K1 500 µg daily	68 ± 6	238	34	200	Mean change (95% CI) Agatston Score (HU)	Coronary Artery	unadjusted: 27 (15, 38) *fully adjusted: 28 (16, 39)*		0.26 *0.34*
		Multivitamin control	68 ±5	236	39	188			unadjusted: 37 (24, 50) *fully adjusted: 36 (27, 47)*		
Shea per protocol analysis	36	Vitamin K1 500 µg daily	68 ± 6	238	34	149	Mean change (95% CI) Agatston Score (HU)	Coronary Artery	unadjusted: 17 (4, 29) *fully adjusted: 24 (2, 46)*		0.03 *0.04*
		Multivitamin control	68 ±5	236	39	146			unadjusted: 37 (24, 50) *fully adjusted: 36 (24, 49)*		
Zwakenberg, [20]	6	MK-7 360 µg daily	69.1 ±8.4	35	2	33	Mean change ± SD in Target to Background Ratio (TBR) of ^18^F-NaF PET Uptake	Femoral Artery Wall	0.09 ±0.55		0.06
		Placebo	69.1 ±8.4	33	6	27			−0.15 ±0.44		
		MK-7 360 µg daily	69.1 ±8.4	35	2	33	Median change (IQR) in CT-based log-transformed calcification		19.7 (2.2–51.4)		0.18
		Placebo	69.1 ±8.4	33	6	27			4.3 (0.1–20.4)		

**Table 5:** Effect of vitamin K supplementation on measures of atherosclerosis.

Author, Analysis	Follow Up (mo)	Study Arms	Age ± SD	Randomized (*n*)	Lost to Follow Up (*n*)	Included in Analysis (*n*)	Outcome Metric	Artery Location	Outcome Measurements		*p* Value of Treatment Effect
									Baseline	End of Follow Up	
Braam [12]	36	MDK	55.4 ± 2.8	66	14	38	Mean (SD) Change in CIMT(mm)	Carotid		0.06 ± 0.06	NS
		MD	55.9 ± 2.8	61	19	30				0.02 ± 0.09	
		Placebo	54.1 ± 3.0	61	5	40				0.05 ± 0.08	
		MDK	55.4 ± 2.8	66	14	38	Mean % Change in CIMT (mm)			9.8%	NS
		MD	55.9 ± 2.8	61	19	30				4.0%	
		Placebo	54.1 ± 3.0	61	5	40				8.6%	
Fulton [15]	6	MK-7	76.0 ± 4.4	40	2	38	CIMT (mm ±SD)	Carotid	0.077 ± 0.015	0.076 ± 0.020	NS
		Placebo	77.1 ± 4.8	40	1	39			0.080 ± 0.021	0.080 ± 0.010	
		MK-7	76.0 ± 4.4	40	2	38	Mean (SD) FMD (%)	Brachial	6.3 ± 2.7	7.6 ± 2.7	NS
		Placebo	77.1 ± 4.8	40	1	39			7.3 ± 2.4	8.6 ± 2.4	
Kurnatowska [17]	9	MK-7/VD^3^	NR	29	1	28	Mean (SD) Change CIMT (mm)	Carotid		0.06 ± 0.08	NS
		VD	NR	13	1	12				0.136 ± 0.05	
		MK-7/VD	NR	29	1	28	Mean (SD) % Change in CIMT (mm)			6.0% ± 7.1%	NS
		VD	NR	13	1	12				13.8% ± 4.9%	

CIMT—carotid-intima media thickness; NR—not reported; NS—not significant. MD: supplement containing 500 mg of calcium, 10 mg of zinc, 150 mg of magnesium and 8 μg of vitamin D3; MDK: supplement containing 500 mg of calcium, 10 mg of zinc, 150 mg of magnesium, 8 μg vitamin D3, 1 mg of vitamin K1; VD: Vitamin D3 10 μg daily

**Table 6:** Effect of vitamin K supplementation on arterial stiffness outcomes.

Author, Analysis Type	Follow-up Period (mos)	Study Arms	Number of Patients Randomized (*n*)	Number Lost to Follow-up (*n*)	Number Included in Final Analysis (*n*)	Outcome Metric	Location of Measurement	Outcome Measurements		*p* Value of Treatment Effect
								Baseline	End of Follow Up	
Fulton, ITT with adjusted baseline [15]	6	MK-7 100 µg daily	40	2	38	PWV (m/s) ± SD	Carotid-Radial PWV (crPWV)	9.7 ± 2.1	9.9 ± 1.4	0.15
		Placebo	40	1	39			10.7 ± 2.3	10.9 ± 3.0	
Knapen [16]	36	MK-7 180 µg daily	120	9	111	Mean change in Stiffness Index β ± SD	Carotid Artery	11.4 ± 3.1	−0.67 ± 2.78	0.018
		Placebo	124	12	112			11.3 ± 4.5	0.15 ± 2.51	
		MK-7 180 µg daily	120	9	111	Mean change in PWV (m/s) ± SD	Carotid-Femoral PWV (cfPWV)	9.9 ± 1.9	−0.36 ± 1.48	0.040
		Placebo	124	12	112			9.7 ± 1.7	+0.021 ± 1.22	
		MK-7 180 µg daily	120	9	111	Mean change in PWV (m/s) ± SD	Carotid-radial PWV (crPWV)	10.2 ± 1.4	−0.44 ± 1.42	0.073
		Placebo	124	12	112			10.1 ± 1.4	−0.091 ± 1.51	
		MK-7 180 µg daily	120	9	111	Mean change in PWV (m/s) ± SD	Local carotid PWV (cPWV)	8.2 ± 1.2	−0.37 ± 1.15	0.19
		Placebo	124	12	112			8.1 ± 1.6	−0.19 ± 1.04	
De Vriese [14]	18	Warfarin (target INR 2–3)	44	27	44	PWV (IQR) (m/s)	Carotid-Femoral PWV (cfPWV)	12.7 (10.4–14.2)	NR	0.56
		Rivaroxaban 10 mg daily	46	22	46			14.6 (12.1–16.1)		
		Rivaroxaban 10 mg daily plus MK-7 2 mg thrice weekly	41	20	42			13.1 (10.6–15.6)		

PWV—pulse wave velocity.

**Table 7:** Effect of vitamin K supplementation on the change in MGP.

Author, Analysis Conducted	Biomarker of Interest	Type of Assay and Source	Metric of Outcome	Study Arms	Baseline	End of Follow Up	* *p* Value	** *p* Value
Brandenburg [13]	Dp-uc-MGP (pmol/L)	IDS, Boldon, UK	Mean ± SD	Vitamin K1	432 ± 149	243 ± 165	<0.001	
				Placebo	483 ± 215	515 ± 276	0.10	
	Change in Dp-uc-MGP (pmol/L)	IDS, Boldon, UK	Mean ± SD	Vitamin K1	N/A	−199 ± 233	N/A	<0.001
				Placebo	N/A	37 ± 112	N/A	
De Vriese [14]	dp-uc-MGP (pmol/L)	Automated assay, Ina K tif MGP iSYS kit; IDS, Boldon, UK	Median [IQR]	Rivaroxaban + vitamin K2	1598 (1058,3324)	853 (707, 1176)	0.04	<0.001
				Rivaroxaban	1632 (1083,2390)	981 (729, 1453)	0.04	
				VKAs	1983 (1486,3087)	2967 (1982, 4737)	0.03	
Fulton [15]	dp-uc-MGP (pmol/L)	ELISA, InaKtif MGP IDS-iSYS assay	Mean ± SD, change from baseline	MK-7	789 ± 363	−130 ± 117		<0.001
				Placebo n	823 ± 360	+13 ± 180		
Knapen [16]	Plasma dp-uc-MGP (pmol/L)	InaKtif MGP iSYS kit, IDS, Boldon, UK	Mean ± SD, change from baseline	MK-7	511 ± 236	−188 ± 157		<0.0001
				Placebo	538 ± 293	+74 ± 182		
Kurnatowska [17]	MGP (pg/mL)	Sandwich enzyme immunoassay, ELISA; USCN Life Science Inc.	Mean ± SD	Vitamin K + D	639.6 ± 187	742.8 ± 249.1	0.06	0.1
				Vitamin D	640.7 ± 195.4	615 ± 165.9	0.6	
	dp-uc-MGP (pmol/L)	inaK-tif MGP iSYS kit, Immunodiagnostic systems	Mean ± SD	Vitamin K + D	1077.1 ± 507.7	961.5 ± 506.7	0.02	0.5
				Vitamin D	793.9 ± 400.3	820.7 ± 565.2	0.7	
Oikonomaki [18]	Serum uc-MGP (ng/mL)	ELISA kit, Cusabio,	Median ± IQR	MK-7	8342 ± 10,047	4218 ± 6505	0.007	0.03
				Standard Care	8903 ± 10,517	8050 ± 12,155	NS	
Shea [19]	MGP (ng/mL)	Radioimmunoassay	Mean ± SD	Vitamin K1	200 ± 48	207 ± 63	NS	
				Control	200 ± 48	192 ± 59	NS	
	MGP (ng/mL)	Radioimmunoassay	Mean (95% CI) 3-year change	Vitamin K1	N/A	7.2 (−1.2, 15.5)	N/A	0.02
				Control		−7.3 (−16.5, 1.8)		
Zwakenberg [20]	dp-uc-MGP (pmol/L)	Sandwich ELISA method, IDS automated analyser IDS-iSYS InaKtif MGP assay	Median (IQR)	MK-7	613 (513–684)	416 (370–552)	*p* < 0.01	
				Placebo	615 (489–743)	645 (552–734)		

* *p* value for within treatment groups; ** *p* value for between treatment groups; Dp-ucMGP—dephosphorylated uncarboxylated Matrix Gla Protein.

**Table 8:** Frequency and type of adverse events experienced by trial participants.

Author	% of Adverse Events Experienced in Treatment Arm	% of Adverse Events Experienced in Control Arm(s)		Adverse Effects Reported	Adverse Effects Reported from Control Group(s)
Braam [12]	4.5	6.6 *	0	Mild Constipation	Mild Constipation
Brandenburg [13]	NR	NR			
De Vriese [14]	0	0	0		
Fulton [15]	45 reported, from 40 patients	27 reported, from 40 patients		Cardiovascular (6), Pain (4), Gastrointestinal Disturbances (10), Postural Symptoms (2), Falls (17), Infections (6)	Cardiovascular (5), Pain (3), Gastrointestinal Disturbances (5), Postural Symptoms (2), Falls (7), Infections (5)
Knapen [16]	NR	NR			
Kurnatowska [17]	3.4	0		Bowel Discomfort	
Oikonomaki [18]	0	0			
Shea [19]	0	0			
Zwakenberg [20]	NR	NR			

* multivitamin group; NR—not reported.

## Appendix A

Search Strategy

Embase Classic + Embase 1947 to 2020 April 28

1. exp vitamin k group/
2. vitamin k$1.ti,ab,kw.
3. acetomenaphthone.ti,ab,kw.
4. farnoquinone.ti,ab,kw.
5. menadiol.ti,ab,kw.
6. menadione.ti,ab,kw.
7. menaquinone.ti,ab,kw.
8. menatetrenone.ti,ab,kw.
9. phytomenadione.ti,ab,kw.
10. 1 or 2 or 3 or 4 or 5 or 6 or 7 or 8 or 9
11. heart muscle ischemia/
12. exp cerebrovascular accident/
13. exp peripheral occlusive artery disease/
14. exp peripheral vascular disease/
15. arterial wall thickness/
16. exp heart hypertrophy/
17. exp calcification/
18. arterial stiffness/
19. pulse wave/
20. pulse pressure/
21. exp mortality/
22. heart death/
23. mortalit*.ti,ab,kw.
24. (sudden cardiac adj2 (death* or arrest*)).ti,ab,kw.
25. ((cardiac or heart) adj2 death*).ti,ab,kw.
26. calcification.ti,ab,kw.
27. calcinosis.ti,ab,kw.
28. calciphylaxis.ti,ab,kw.
29. calcific uremic arteriopathy.ti,ab,kw.
30. ((blood vessel* or aort* or arter* or vacular) adj2 stiff*).ti,ab,kw.
31. pulse wave.ti,ab,kw.
32. pulse pressure.ti,ab,kw.
33. arterial pressure wave.ti,ab,kw.
34. arter* pulsation.ti,ab,kw.
35. arter* pulse curve.ti,ab,kw.
36. ((myocard* or heart or cardiac or coronary or subendocardial) adj3 (attack* or isch?emi* or hypoxi* or anoxia)).ti,ab,kw.
37. stroke.ti,ab,kw.
38. cerebrovascular accident*.ti,ab,kw.
39. isch?emic cerebral attack*.ti,ab,kw.
40. (cerebr* vascular adj2 (accident* or insufficienc*)).ti,ab,kw.
41. (cerebrovascular adj2 (accident* or injur* or arrest* or failure* or insufficienc*)).ti,ab,kw.
42. (brain adj2 (attack* or insult* or accident*)).ti,ab,kw.
43. ((ventricular or cardiac or heart or myocard*) adj3 hypertroph*).ti,ab,kw.
44. ventricular wall thickness.ti,ab,kw.
45. atherosclerosis.ti,ab,kw.
46. intima* media thickness.ti,ab,kw.
47. carotidintima media thickness.ti,ab,kw.
48. ((aort* or arter*) adj2 thickness).ti,ab,kw.
49. or/11–48
50. 10 and 49
51. Randomized controlled trial/
52. Controlled clinical study/
53. random$.ti,ab.
54. randomization/
55. intermethod comparison/
56. placebo.ti,ab.
57. (compare or compared or comparison).ti.
58. ((evaluated or evaluate or evaluating or assessed or assess) and (compare or compared or comparing or comparison)).ab.
59. (open adj label).ti,ab.
60. double blind procedure/
61. parallel group$1.ti,ab.
62. (crossover or cross over).ti,ab.
63. ((assign$ or match or matched or allocation) adj5 (alternate or group$1 or intervention$1 or patient$1 or subject$1 or participant$1)).ti,ab.
64. (assigned or allocated).ti,ab.
65. (controlled adj7 (study or design or trial)).ti,ab.
66. (volunteer or volunteers).ti,ab.
67. human experiment/
68. trial.ti.
69. or/51–68
70. (random$ adj sampl$).mp. adj7 ((cross section$ or questionnaire$1 or survey$ or database$1).ti,ab. not (comparative study/or controlled study/or randomi?ed controlled.ti,ab. or randomly assigned.ti,ab.)) [mp = title, abstract, heading word, drug trade name, original title, device manufacturer, drug manufacturer, device trade name, keyword, floating subheading word, candidate term word]
71. Cross-sectional study/not (randomized controlled trial/or controlled clinical study/or controlled study/or randomi?ed controlled.ti,ab. or control group$1.ti,ab.)
72. (((case adj control$) and random$) not randomi?ed controlled).ti,ab.
73. (Systematic review not (trial or study)).ti.
74. (nonrandom$ not random$).ti,ab.
75. Random field$.ti,ab.
76. (random cluster adj3 sampl$).ti,ab.
77. (review.ab. and review.pt.) not trial.ti.
78. we searched.ab. and (review.ti. or review.pt.)
79. update review.ab.
80. (databases adj4 searched).ab.
81. (rat or rats or mouse or mice or swine or porcine or murine or sheep or lambs or pigs or piglets or rabbit or rabbits or cat or cats or dog or dogs or cattle or bovine or monkey or monkeys or trout or marmoset$1).ti. and animal experiment/
82. Animal experiment/not (human experiment/or human/)
83. or/70–82
84. 69 not 83
85. 50 and 84
86. 85 not (review/not (Randomized controlled trial/or Controlled clinical study/))

Ovid MEDLINE(R), Ovid MEDLINE(R) Daily and Epub Ahead of Print, In-Process & Other Non-Indexed Citations 1946 to Present

1. exp Vitamin K/
2. vitamin k$1.ti,ab,kw,kf.
3. acetomenaphthone.ti,ab,kw,kf.
4. farnoquinone.ti,ab,kw,kf.
5. menadiol.ti,ab,kw,kf.
6. menadione.ti,ab,kw,kf.
7. menaquinone.ti,ab,kw,kf.
8. menatetrenone.ti,ab,kw,kf.
9. phytomenadione.ti,ab,kw,kf.
10. 1 or 2 or 3 or 4 or 5 or 6 or 7 or 8 or 9
11. exp Stroke/
12. peripheral arterial disease/
13. atherosclerosis/
14. arterial occlusive diseases/
15. exp Peripheral Vascular Diseases/
16. exp Cardiomegaly/
17. exp vascular calcification/
18. calcinosis/
19. Vascular Stiffness/
20. exp Pulse Wave Analysis/
21. exp Mortality/
22. exp Myocardial Infarction/
23. Calciphylaxis/
24. exp Cerebrovascular Disorders/
25. exp Myocardial Ischemia/
26. mortalit*.ti,ab,kw,kf.
27. (sudden cardiac adj2 (death* or arrest*)).ti,ab,kw,kf.
28. ((cardiac or heart) adj2 death*).ti,ab,kw,kf.
29. calcification.ti,ab,kw,kf.
30. calcinosis.ti,ab,kw,kf.
31. calciphylaxis.ti,ab,kw,kf.
32. calcific uremic arteriopathy.ti,ab,kw,kf.
33. ((blood vessel* or aort* or arter* or vacular) adj2 stiff*).ti,ab,kw,kf.
34. pulse wave.ti,ab,kw,kf.
35. pulse pressure.ti,ab,kw,kf.
36. arterial pressure wave.ti,ab,kw,kf.
37. arter* pulsation.ti,ab,kw,kf.
38. arter* pulse curve.ti,ab,kw,kf.
39. ((myocard* or heart or cardiac or coronary or subendocardial) adj3 (attack* or isch?emi* or hypoxi* or anoxia)).ti,ab,kw,kf.
40. stroke.ti,ab,kw,kf.
41. cerebrovascular accident*.ti,ab,kw,kf.
42. isch?emic cerebral attack*.ti,ab,kw,kf.
43. (cerebr* vascular adj2 (accident* or insufficienc*)).ti,ab,kw,kf.
44. (cerebrovascular adj2 (accident* or injur* or arrest* or failure* or insufficienc*)).ti,ab,kw,kf.
45. (brain adj2 (attack* or insult* or accident*)).ti,ab,kw,kf.
46. ((ventricular or cardiac or heart or myocard*) adj3 hypertroph*).ti,ab,kw,kf.
47. ventricular wall thickness.ti,ab,kw,kf.
48. atherosclerosis.ti,ab,kw,kf.
49. intima* media thickness.ti,ab,kw,kf.
50. carotidintima media thickness.ti,ab,kw,kf.
51. ((aort* or arter*) adj2 thickness).ti,ab,kw,kf.
52. or/11–51
53. randomized controlled trial.pt.
54. controlled clinical trial.pt.
55. random*.ti,ab,kw,kf.
56. placebo.ab.
57. drug therapy.fs.
58. groups.ab.
59. trial.ti,ab.
60. 53 or 54 or 55 or 56 or 57 or 58 or 59
61. exp animals/not humans.sh.
62. 60 not 61
63. 10 and 52 and 62
64. limit 63 to “review”
65. 63 not 64

EBM Reviews—Cochrane Central Register of Controlled Trials

1. exp Vitamin K/
2. vitamin k$1.ti,ab,kw,kf.
3. acetomenaphthone.ti,ab,kw,kf.
4. farnoquinone.ti,ab,kw,kf.
5. menadiol.ti,ab,kw,kf.
6. menadione.ti,ab,kw,kf.
7. menaquinone.ti,ab,kw,kf.
8. menatetrenone.ti,ab,kw,kf.
9. phytomenadione.ti,ab,kw,kf.
10. 1 or 2 or 3 or 4 or 5 or 6 or 7 or 8 or 9
11. exp Stroke/
12. peripheral arterial disease/
13. atherosclerosis/
14. arterial occlusive diseases/
15. exp Peripheral Vascular Diseases/
16. exp Cardiomegaly/
17. exp vascular calcification/
18. calcinosis/
19. Vascular Stiffness/
20. exp Pulse Wave Analysis/
21. exp Mortality/
22. exp Myocardial Infarction/
23. Calciphylaxis/
24. exp Cerebrovascular Disorders/
25. exp Myocardial Ischemia/
26. mortalit*.ti,ab,kw,kf.
27. (sudden cardiac adj2 (death* or arrest*)).ti,ab,kw,kf.
28. ((cardiac or heart) adj2 death*).ti,ab,kw,kf.
29. calcification.ti,ab,kw,kf.
30. calcinosis.ti,ab,kw,kf.
31. calciphylaxis.ti,ab,kw,kf.
32. calcific uremic arteriopathy.ti,ab,kw,kf.
33. ((blood vessel* or aort* or arter* or vacular) adj2 stiff*).ti,ab,kw,kf.
34. pulse wave.ti,ab,kw,kf.
35. pulse pressure.ti,ab,kw,kf.
36. arterial pressure wave.ti,ab,kw,kf.
37. arter* pulsation.ti,ab,kw,kf.
38. arter* pulse curve.ti,ab,kw,kf.
39. ((myocard* or heart or cardiac or coronary or subendocardial) adj3 (attack* or isch?emi* or hypoxi* or anoxia)).ti,ab,kw,kf.
40. stroke.ti,ab,kw,kf.
41. cerebrovascular accident*.ti,ab,kw,kf.
42. isch?emic cerebral attack*.ti,ab,kw,kf.
43. (cerebr* vascular adj2 (accident* or insufficienc*)).ti,ab,kw,kf.
44. (cerebrovascular adj2 (accident* or injur* or arrest* or failure* or insufficienc*)).ti,ab,kw,kf.
45. (brain adj2 (attack* or insult* or accident*)).ti,ab,kw,kf.
46. ((ventricular or cardiac or heart or myocard*) adj3 hypertroph*).ti,ab,kw,kf.
47. ventricular wall thickness.ti,ab,kw,kf.
48. atherosclerosis.ti,ab,kw,kf.
49. intima* media thickness.ti,ab,kw,kf.
50. carotidintima media thickness.ti,ab,kw,kf.
51. ((aort* or arter*) adj2 thickness).ti,ab,kw,kf.
52. or/11–51
53. 10 and 52

Web of Science

Indexes = SCI-EXPANDED, SSCI, A&HCI, CPCI-S, CPCI-SSH, ESCI Timespan = All years

#1 #22 AND #15
#2 #21 OR #20 OR #19 OR #18 OR #17 OR #16
#3 TOPIC: (evaluat* and compar*) OR TOPIC: (assess* and compar*)
#4 TOPIC: (allocation NEAR/5 alternate) OR TOPIC: (allocation NEAR/5 group*) OR TOPIC: (allocation NEAR/5 intervention*) OR TOPIC: (allocation NEAR/5 patient*) OR TOPIC: (allocation NEAR/5 subject*) OR TOPIC: (allocation NEAR/5 participant*)
#5 TOPIC: (match* NEAR/5 alternate) OR TOPIC: (match* NEAR/5 group*) OR TOPIC: (match* NEAR/5 intervention*) OR TOPIC: (match* NEAR/5 patient*) OR TOPIC: (match* NEAR/5 subject*) OR TOPIC: (match* NEAR/5 participant*)
#6 TOPIC: (assign* NEAR/5 alternate) OR TOPIC: (assign* NEAR/5 group*) OR TOPIC: (assign* NEAR/5 intervention*) OR TOPIC: (assign* NEAR/5 patient*) OR TOPIC: (assign* NEAR/5 subject*) OR TOPIC: (assign* NEAR/5 participant*)
#7 TOPIC: (controlled NEAR/7 study) OR TOPIC: (controlled NEAR/7 design) OR TOPIC: (controlled NEAR/7 trial)
#8 TOPIC: (random*) OR TOPIC: (placebo) OR TITLE: (compare or compared or comparison) OR TOPIC: (open NEAR/1 label) OR TOPIC: (parallel group*) OR TOPIC: (crossover or cross over) OR TOPIC: (assigned) OR TOPIC: (allocated) OR TOPIC: (volunteer) OR TOPIC: (volunteers)
#9 #14 AND #1
#10 #13 OR #12 OR #11 OR #10 OR #9 OR #8 OR #7 OR #6 OR #5 OR #4 OR #3 OR #2
#11 TOPIC: (aort* NEAR/2 thickness) OR TOPIC: (arter* NEAR/2 thickness)
#12 TOPIC: (myocard* NEAR/3 hypertroph*) OR TOPIC: (heart NEAR/3 hypertroph*) OR TOPIC: (cardiac NEAR/3 hypertroph*) OR TOPIC: (ventricular NEAR/3 hypertroph*)
#13 TOPIC: (brain NEAR/2 attack*) OR TOPIC: (brain NEAR/2 insult*) OR TOPIC: (brain NEAR/2 accident*)
#14 TOPIC: (cerebrovascular NEAR/2 accident*) OR TOPIC: (cerebrovascular NEAR/2 injur*) OR TOPIC: (cerebrovascular NEAR/2 arrest*) OR TOPIC: (cerebrovascular NEAR/2 failure*) OR TOPIC: (cerebrovascular NEAR/2 insufficienc*)
#15 TOPIC: (cerebr* vascular NEAR/2 accident*) OR TOPIC: (cerebr* vascular NEAR/2 insufficienc*)
#16 TOPIC: (myocard* NEAR/3 anoxia) OR TOPIC: (heart NEAR/3 anoxia) OR TOPIC: (cardiac NEAR/3 anoxia) OR TOPIC: (coronary NEAR/3 anoxia) OR TOPIC: (subendocardial NEAR/3 anoxia)
#17 TOPIC: (myocard* NEAR/3 hypoxi*) OR TOPIC: (heart NEAR/3 hypoxi*) OR TOPIC: (cardiac NEAR/3 hypoxi*) OR TOPIC: (coronary NEAR/3 hypoxi*) OR TOPIC: (subendocardial NEAR/3 hypoxi*) OR TOPIC: (myocard* NEAR/3 ischaemi*) OR TOPIC: (heart NEAR/3 ischaemi*) OR TOPIC: (cardiac NEAR/3 ischaemi*) OR TOPIC: (coronary NEAR/3 ischaemi*) OR TOPIC: (subendocardial NEAR/3 ischaemi*)
#18 TOPIC: (myocard* NEAR/3 attack*) OR TOPIC: (heart NEAR/3 attack*) OR TOPIC: (cardiac NEAR/3 attack*) OR TOPIC: (coronary NEAR/3 attack*) OR TOPIC: (subendocardial NEAR/3 attack*) OR TOPIC: (myocard* NEAR/3 ischemi*) OR TOPIC: (heart NEAR/3 ischemi*) OR TOPIC: (cardiac NEAR/3 ischemi*) OR TOPIC: (coronary NEAR/3 ischemi*) OR TOPIC: (subendocardial NEAR/3 ischemi*)
#19 TOPIC: (blood vessel* NEAR/2 stiff*) OR TOPIC: (vacular NEAR/2 stiff*) OR TOPIC: (arter* NEAR/2 stiff*) OR TOPIC: (aort* NEAR/2 stiff*)
#20 TOPIC: (cardiac NEAR/2 death*) OR TOPIC: (heart NEAR/2 death*)
#21 TOPIC: (sudden cardiac NEAR/2 death*) OR TOPIC: (sudden cardiac NEAR/2 arrest*)
#22 TOPIC: (mortalit*) OR TOPIC: (calcification) OR TOPIC: (calcinosis) OR TOPIC: (calciphylaxis) OR TOPIC: (calcific uremic arteriopathy) OR TOPIC: (pulse wave) OR TOPIC: (pulse pressure) OR TOPIC: (arterial pressure wave) OR TOPIC: (arter* pulsation) OR TOPIC: (arter* pulse curve) OR TOPIC: (stroke) OR TOPIC: (cerebrovascular accident*) OR TOPIC: (ischemic cerebral attack*) OR TOPIC: (ischaemic cerebral attack*) OR TOPIC: (ventricular wall thickness) OR TOPIC: (atherosclerosis) OR TOPIC: (intima* media thickness) OR TOPIC: (carotidintima media thickness)
#23 TOPIC: (vitamin k) OR TOPIC: (acetomenaphthone) OR TOPIC: (farnoquinone) OR TOPIC: (menadiol) OR TOPIC: (menadione) OR TOPIC: (menaquinone) OR TOPIC: (menatetrenone) OR TOPIC: (phytomenadione) OR TOPIC: (vitamin K1) OR TOPIC: (vitamin K2) OR TOPIC: (vitamin K3)
